# Roll-your-own tobacco use among Canadian youth: is it a bigger problem than we think?

**DOI:** 10.1186/1471-2458-12-557

**Published:** 2012-07-27

**Authors:** Scott T Leatherdale, Robin Burkhalter

**Affiliations:** 1 School of Public Health and Health Systems, University of Waterloo, 200 University Avenue, Waterloo, ON, Canada, N2L 3G1; 2 Propel Centre for Population Health Impact, Canadian Cancer Society, University of Waterloo, Waterloo, ON, Canada

**Keywords:** Roll-your-own tobacco, Disposable income, Youth, Drug use, Smoking

## Abstract

**Background:**

Despite the apparent decline in the popularity of roll-your-own (RYO) cigarettes over the past few decades, RYO tobacco products are widely available and used by a substantial number of adult smokers. Considering research has yet to examine the prevalence of RYO tobacco use among youth populations, this manuscript examines the prevalence of RYO tobacco use and factors associated with RYO use in a nationally representative sample of youth smokers from Canada.

**Methods:**

This study used data collected from 3,630 current smokers in grades 9 to 12 as part of the 2008-09 Canadian Youth Smoking Survey (YSS). Descriptive analyses of the sample demographic characteristics, smoking status, cigarettes per day, weekly spending money, and frequency of marijuana use were examined by RYO tobacco ever use and RYO tobacco current use. Two logistic regression models were used to examine factors associated with RYO tobacco ever use and RYO tobacco current use.

**Results:**

We identified that 51.2% of current smokers were RYO ever users and 24.2% were RYO current users. The prevalence of RYO current users was highest in Atlantic Canada (40.1%) and lowest in Quebec (12.3%). RYO current users were more likely to be male (OR 1.27), to be daily smokers (OR 1.75), to use marijuana once a month or more (OR 2.74), and to smoke 11 or more cigarettes per day (OR 6.52). RYO current users were less likely to be in grade 11 (OR 0.65) or grade 12 (OR 0.40) and less likely to have between $20 to $100 (OR 0.44) or more than $100 (OR 0.45) of disposable income.

**Conclusions:**

Developing a better understanding of RYO tobacco use among youth is important for advancing population-level tobacco control prevention strategies and cessation programs. We identified that RYO tobacco use is not a negligible problem among Canadian youth. Ongoing research is needed to continue monitoring the prevalence of RYO use among youth and the factors associated with its use, but to also monitor if this more affordable tobacco product is being targeted to price sensitive youth smokers.

## Background

Roll-your-own cigarettes (RYO) refer to cigarettes that are made by hand or with the assistance of a rolling machine using loose fine-cut tobacco. Despite the apparent decline in the popularity of RYO cigarettes over the past few decades [1,2], RYO tobacco products are widely available and used by a substantial number of adult smokers [2-5]. This is important as evidence suggests that smokers who use RYO tobacco compared to factory made (FM) cigarettes are at a greater risk for cancers of the lung [6], oesophagus [7], and the mouth, pharynx and larynx [8]. Considering research has yet to examine the prevalence of RYO tobacco use among youth populations, it is important to determine the extent to which this tobacco product may warrant additional tobacco control prevention action.

Cigarette taxes are frequently used to discourage smoking since higher cigarette prices are associated with reduced cigarette consumption, especially among youth populations [9,10]. However, in Canada, fine-cut tobacco used to make RYO cigarettes is currently taxed at a lower rate than FM cigarettes making them a cheaper option for youth smokers [11]. In 2008, a smoker in Canada would have to pay $20.67(Can) of Federal tax and between $20.26-$42.00(Can) in Provincial/Territorial tax on 200 manufactured cigarettes, whereas a smoker purchasing 100 g of fine-cut loose-leaf tobacco (~200 RYO cigarettes) would have only had to pay $7.31(Can) in Federal tax and between $10.30-$33.54(Can) in Provincial/Territorial tax (11). These large discrepancies in Federal and Provincial/Territorial taxes are important as price sensitive youth may compensate for price/taxation increases by shifting from FM cigarettes to cheaper RYO cigarettes instead of quitting or reducing consumption [1]. For instance, recent research has identified that in the FM cigarette market, a substantial proportion of Canadian youth smokers now regularly smoke ‘discount’ cigarettes instead of more expensive premium cigarette brands [12]. As such, it would be informative to build on such evidence by examining the prevalence of RYO tobacco use among Canadian youth.

A paucity of research has examined RYO tobacco use, there are no previous national surveillance data with youth in Canada that have measured RYO tobacco use [13], and there appears to be no research even internationally specific to youth populations. Of the limited evidence available from adults, data suggest that RYO smokers tend to be male [2,5,14], to be daily smokers [5], to be heavier smokers [2,3,5,14], to be lower income [2-5], to be drug users [2,4,14], and younger [2,4]. Research also suggests that the reason most frequently reported for using RYO among adult RYO users is the lower cost [4,15]. Research is required to determine if youth RYO smokers exhibit similar characteristics; valuable insight for informing future tobacco control prevention and cessation activities. Considering the 2008 wave of the National Youth Smoking Survey.

## Methods

This study used nationally representative data collected from 3,630 current smokers in grades 9 to 12 as part of the 2008-09 Canadian Youth Smoking Survey (YSS) [16]. In brief, the target population consisted of all young Canadian residents attending public and private secondary schools in 10 Canadian provinces; youth residing in the Yukon, Nunavut and the Northwest Territories were excluded from the target population, as were youth living in institutions or on First Nation Reserves, and youth attending special schools or schools on military bases. Data were collected using a 30-40 minute classroom-based survey of a representative sample of schools and students. School sampling was based on a stratified multistage design to enhance the efficiency (precision) of estimates of population means and proportions, over purely random sampling of units like boards or schools. In each province, schools were then randomly selected to participate with probabilities proportional to the total enrolment in their boards. The number of private schools randomly selected to participate was proportional to the number of students enrolled in private schools in each province compared to the total in public schools. Within each participating school, all students in the survey grades were eligible to participate. Research ethics approval for this study was obtained from the University of Waterloo Human Research Ethics Committee and local institutional review boards where required. In 81% of participating secondary schools, active information with passive consent was used to reduce demands on schools and to increase student participation rates. The researcher informed the parents of the students via mail and asked them to call a toll-free number if they refused their child’s participation. Based on school or board request, in the remaining 19% of secondary schools, active parental permission (signed parental permission for students to participate in the survey) was required. The University of Waterloo Office of Research Ethics and appropriate School Board and Public Health Ethics committees approved all procedures, including passive consent. The YSS survey was administered to students during class time and participants were not provided compensation. Research ethics approval for this study was obtained from the University of Waterloo Human Research Ethics Committee and local institutional review boards where required. The survey design and sample weights allow us to produce population-based estimates within this manuscript. Detailed information on the 2008-09 YSS is available in print [13] and online (http://www.yss.uwaterloo.ca).

Current smokers were respondents who reported that they had smoked at least 100 cigarettes in their lifetime and had smoked at least one whole cigarette in the 30 days preceding the survey. *Ever use* of RYO tobacco was measured by asking current smokers to report if they have ever tried smoking RYO cigarettes. *Current use* of RYO tobacco was measured by asking current smokers to report if they have smoked RYO cigarettes in the previous 30 days. Smoking status among current smokers was measured by asking on how many of the last 30 days they smoked one or more cigarettes. Daily smokers reported smoking all 30 days (everyday) and occasional smokers reported smoking at least one day in the last 30 days but not everyday. The average number of cigarettes per day was measured by asking respondents to report how many cigarettes they usually smoked on days that they smoked in the last 30 days. The YSS also collected information on demographics, weekly spending money, and frequency of marijuana use. Additional details on the 2008-2009 YSS are available online (http://www.yss.uwaterloo.ca).

Descriptive analyses of the sample characteristics were examined by RYO ever use and RYO current use. We then conducted two logistic regression models to examine factors associated with RYO ever use and RYO current use. Survey weights were used for the descriptive statistics to adjust for differential response rates across regions or groups. The statistical package SAS 9.2 was used for all analyses [17].

## Results

### Descriptive statistics

Overall, 51.2% (n = 98,959) of current smokers were RYO ever users and 24.2% (n = 46,710) were RYO current users. Among males, 53.3% (n = 60,599) reported ever RYO use and 25.4% (n = 28,812) reported current RYO use. Among females, 48.1% (n = 38,360) reported ever RYO use and 22.4% (n = 17,898) reported current RYO use. As shown in Table 1, males were more likely than females to be RYO ever users (χ^2^ = 9.7, *df* = 1, p < 0.01) and RYO current users (χ^2^ = 4.1, *df* = 1, p < 0.05). Daily smokers were more likely than occasional smokers to be RYO ever users (χ^2^ = 140.1, *df* = 1, p < 0.001) and RYO current users (χ^2^ = 71.0, *df* = 1, p < 0.001). Compared to current smokers who are not monthly marijuana users, current smokers who also report smoking marijuana once a month or more were more likely to be RYO ever users (χ^2^ = 105.1, *df* = 2, p < 0.001) and RYO current users (χ^2^ = 104.8, *df* = 2, p < 0.001).

**Table 1:** Descriptive statistics examining roll-your-own (RYO) tobacco ever use and current use among current smokers in grades 9 to 12, 2008-2009, Canada

	**Roll-your-own Tobacco Ever Use**		**Roll-your-own Tobacco Current Use**	
	**Yes (n = 98,959) %**^**a**^	**No (n = 94,497) %**^**a**^	**Yes (n = 46,710) %**^**a**^	**No (n = 146,746) %**^**a**^
Sex				
Female	38.8	43.9	38.3	42.2
Male	61.2	56.1	61.7	57.8
Grade				
9	15.3	15.3	17.7	14.5
10	23.1	28.0	30.1	24.1
11	31.4	25.1	29.8	27.8
12	30.2	31.6	22.4	33.6
Smoking status				
Current daily smoker	61.7	42.0	64.5	48.1
Current occasional smoker	38.3	58.0	35.5	51.9
Average number of cigarettes per day				
A few puffs or 1 cigarette	4.2	13.3	2.7	10.5
2 or 3 cigarettes	19.9	23.3	18.1	22.6
4 to 10 cigarettes	48.3	44.1	46.2	46.3
11 or more cigarettes	27.6	19.3	33.0	20.6
Frequency of marijuana use				
Never used marijuana	5.0	14.6	3.6	11.6
Has tried but does not use monthly	16.7	19.6	10.4	20.5
Uses once a month or more	78.3	65.8	86.0	67.9
Weekly spending money				
$0	9.1	7.9	12.3	7.3
$1 to $20	21.8	23.1	24.5	21.7
$21 to $100	38.7	35.0	33.8	37.9
More than $100	30.4	34.0	29.4	33.1

^a^ weighted population estimate.

# not reportable due to small cell size.

**Figure 1:**
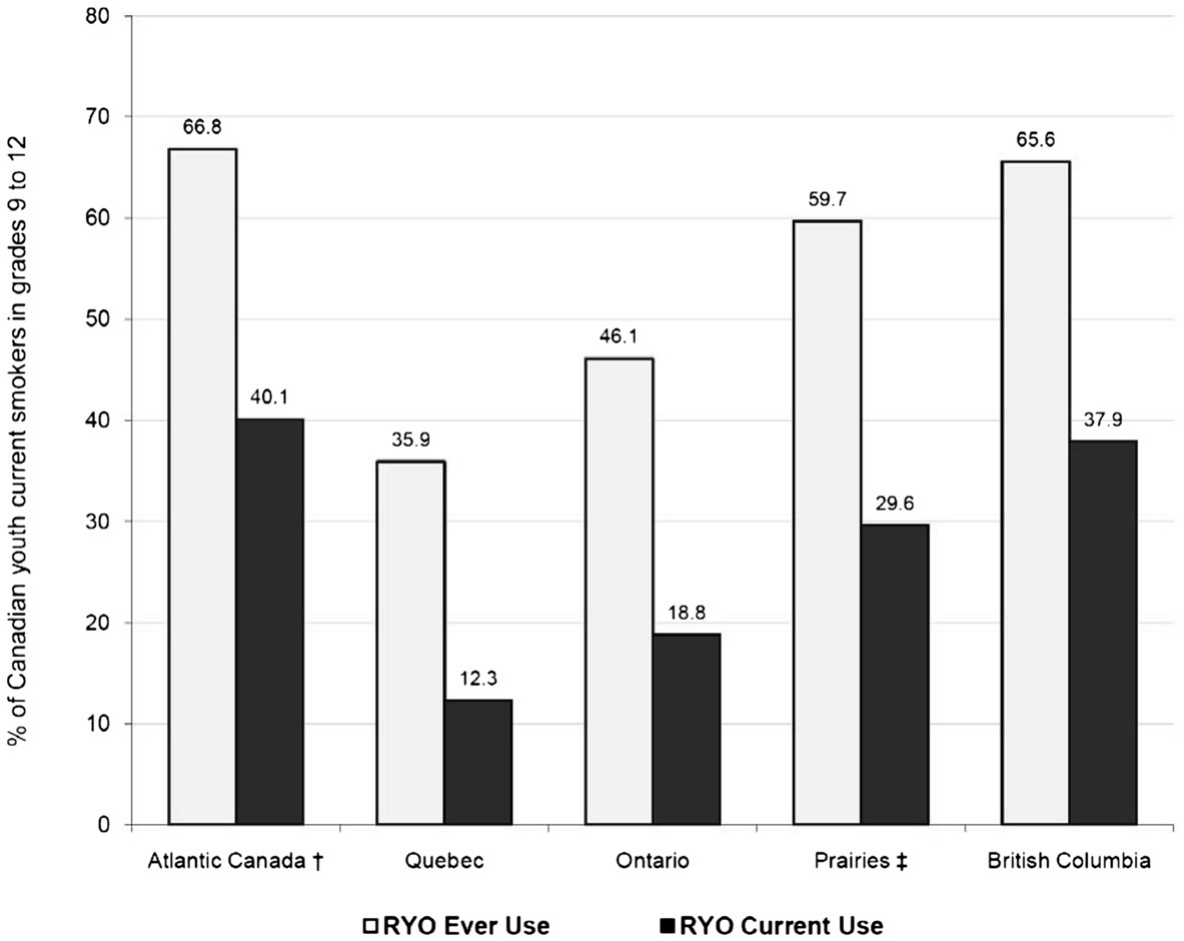
**Prevalence of roll-your-own (RYO) tobacco use by region of Canada (Canada, 2008-2009)**.

**Figure 2:**
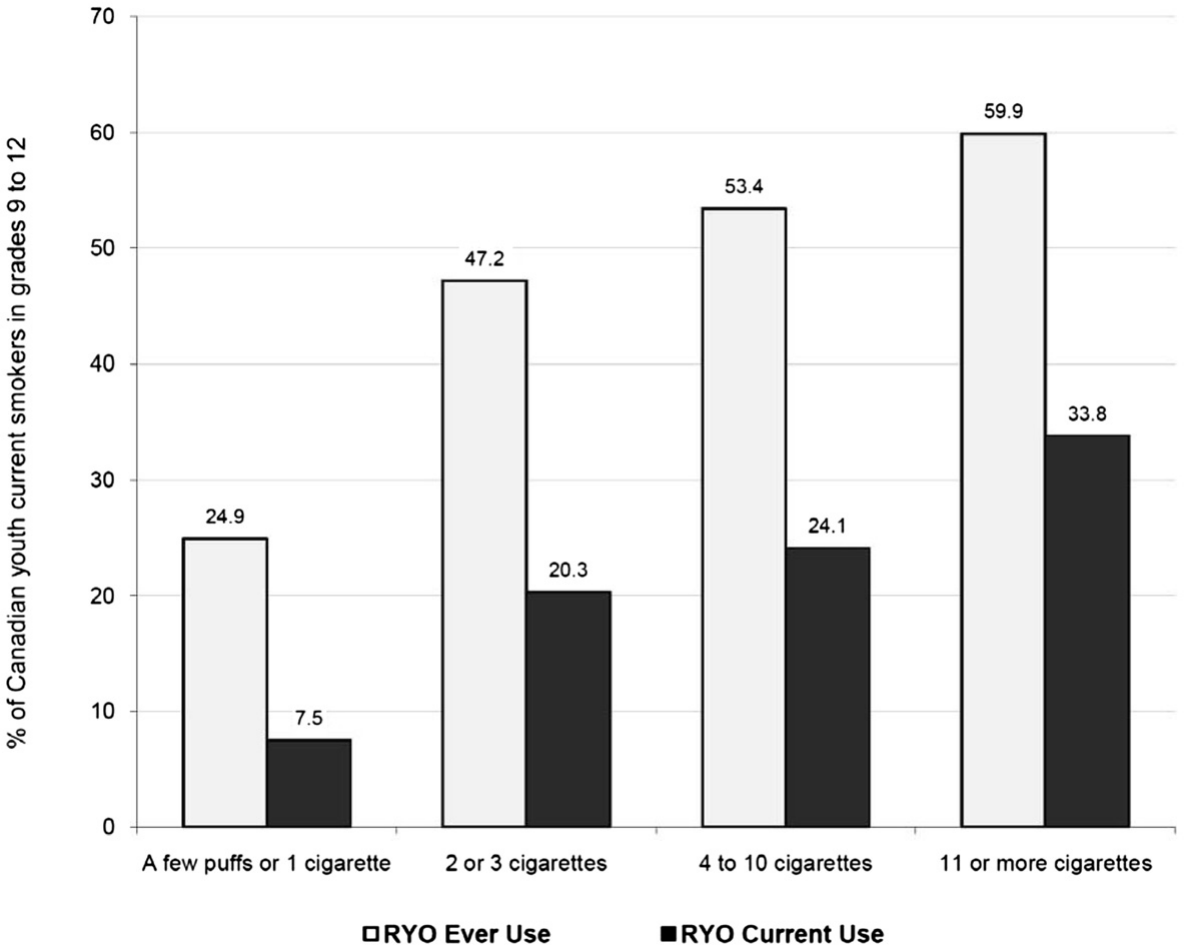
**Prevalence of roll-your-own (RYO) tobacco use by average number of cigarettes per day (Canada, 2008-2009)**.

As shown in Figure 1, there were some substantial regional differences in RYO ever and RYO current use. For instance, the highest rates of RYO current use were reported in Atlantic Canada (40.1%) and British Columbia (37.9%), whereas Quebec had the lowest reported rate of RYO current use (12.3%). As shown in Figure 2, the prevalence of RYO ever use and RYO current use both increased as the average number of cigarettes per day increased. For instance, over a third (33.8%) of smokers who report smoking 11 or more cigarettes per day reported currently using RYO compared to only one in five (20.3%) smokers who reported smoking 2 to 3 cigarettes per day.

### Factors associated with RYO ever use

As shown in Table 2, male students were more likely than female students to report RYO ever use. Daily smokers were more likely than occasional smokers to report RYO ever use. Similarly, compared to students who have never used marijuana, students were more likely to be RYO ever users if they have tried marijuana. Students were less likely to report RYO ever use if they had more than $100 of weekly spending money. Students in grade 12 were less likely to report RYO ever use relative to students in grade 9.

**Table 2:** Logistic regression analyses examining characteristics associated with roll-your-own (RYO) tobacco ever use and current use among current smokers in grades 9 to 12, 2008-2009, Canada

**Adjusted Odds Ratio (95% CI)**^**§**^		
	**Roll-your-own Tobacco Ever Use**^*a*^	**Roll-your-own Tobacco Current Use**^*b*^
Sex		
Female	1.00	1.00
Male	1.39 (1.18, 1.62)***	1.27 (1.05, 1.53)*
Grade		
9	1.00	1.00
10	0.80 (0.62, 1.03)	0.92 (0.69, 1.23)
11	1.02 (0.80, 1.31)	0.65 (0.48, 0.86)**
12	0.62 (0.48, 0.81)***	0.40 (0.29, 0.54)***
Smoking status		
Current occasional smoker	1.00	1.00
Current daily smoker	2.35 (2.01, 2.74)***	1.75 (1.40, 2.19)***
Frequency of marijuana use		
Never used marijuana	1.00	1.00
Has tried but does not use monthly	1.72 (1.23, 2.40)**	1.56 (0.95, 2.54)
Uses once a month or more	2.24 (1.67, 2.99)***	2.74 (1.79, 4.20)***
Weekly spending money		
$0	1.00	1.00
$1 to $20	0.89 (0.65, 1.22)	0.67 (0.47, 0.96)*
$21 to $100	0.91 (0.67, 1.22)	0.44 (0.31, 0.61)***
More than $100	0.68 (0.50, 0.92)*	0.45 (0.31, 0.64)***
Average number of cigarettes per day		
A few puffs or 1 cigarette		1.00
2 or 3 cigarettes		3.61 (2.10, 6.21)***
4 to 10 cigarettes		4.82 (2.82, 8.25)***
11 or more cigarettes		6.52 (3.72, 11.40)***
***c*** statistic	*0.681*	*0.727*

Note: § Odds ratios adjusted for all other variables in the table and controlling for Region.

^a^ 1 = Ever smoked RYO tobacco (n = 1,691), 0 = Never smoked RYO tobacco (n = 1,284).

^b^ 1 = Currently smokes RYO tobacco (n = 936), 0 = Does not currently smoke RYO tobacco (n = 2,004).

^*^p < .05 ^**^p < .01 ^***^p < .001.

### Factors associated with RYO current use

As shown in Table 2, male students were more likely than female students to report RYO current use. Daily smokers were more likely than occasional smokers to report RYO current use. Similarly, compared to students who have never used marijuana, students were more likely to be RYO current users if they use marijuana monthly. Students were more likely to be RYO current users if they reported smoking more than 1 cigarette per day. Students were less likely to report RYO current use if they had any weekly spending money relative to students with no weekly spending money. Compared to students in grade 9, students were less likely to report RYO current use if they were in grade 11 or 12.

## Discussion

Developing a better understanding of RYO tobacco use among youth is important for advancing population-level tobacco control prevention strategies and cessation programs. We identified that RYO tobacco use is not a negligible problem among Canadian youth smokers, as almost one in four reported currently using RYO tobacco. The high level of RYO use among Canadian youth is cause for concern, especially when one considers that the prevalence of current RYO use was higher among our sample of youth when compared to samples of the Canadian adult population [2]. Moreover, the surprisingly high prevalence of RYO current users who are female warrants additional study as research has previously suggested that RYO use is dominated by male smokers. It is clear that RYO tobacco prevention and cessation needs to become better integrated into existing surveillance systems and tobacco control initiatives.

To our knowledge, no studies have examined RYO use among youth smokers despite research previously identifying this population as a priority [4]. However, consistent with the adult literature [2,3,5,14], we identified that youth who were heavier and more frequent smokers were more likely to smoke RYO tobacco. This finding is cause for concern as research has previously identified that RYO smokers are not only more addicted, but they are also less likely to make quit attempts [2] or intend to quit in the future [5]. As such, even though RYO smokers represent a smaller portion of the entire smoking population relative to FM cigarette smokers, knowing that they are more frequent and heavier smokers suggests that they may actually be at increased risk for future smoking related morbidity and mortality [6-8]. Additional research is required to tailor appropriate cessation interventions to this high-risk population.

RYO tobacco is not only more affordable than FM cigarettes, but RYO cigarettes can also be rolled thinner than FM cigarettes, saving tobacco and the relative cost per cigarette. This is an important consideration for price sensitive youth smokers, as evidence suggests that even adult smokers will engage in price minimizing behaviours to compensate for increased manufactured cigarette costs, such as purchasing RYO tobacco [15,18]. Consistent with previous research on adults [5], we identified that the disposable income of youth was associated with RYO tobacco use, where RYO tobacco use is more common among youth with less spending money. The importance of income suggests that as long as a discrepancy in the excise tax on FM and fine-cut tobacco exists, smokers may compensate for price increases by shifting from FM to RYO instead of quitting [1]. Not surprisingly, the provinces in Atlantic Canada identified as having the highest prevalence rates of current RYO use (refer to Figure 1), are also the provinces in Canada with the largest difference in the FM:RYO tax ratio in Canada [11]. These data provide evidence that it should be a public health priority to increase the excise taxes (Federal and Provincial/Territorial taxes) on fine-cut tobacco used for RYO cigarettes to make the cost equivalent to FM cigarettes. However, it is likely that an even larger public health benefit would be observed if fine-cut tobacco should be taxed at a higher rate the FM cigarettes to compensate for the ability RYO smokers have to roll thinner cigarettes using less tobacco [4].

Consistent with research among adults showing that the younger age groups are the most likely to use RYO tobacco [4,5], we identified that the likelihood of RYO use decreased by grade. Our finding is likely a function of youth in higher grades reporting more disposable income than youth in lower grades; as such, youth in lower grades are more apt to smoke RYO since it is more affordable. However, when one considers that smoking prevalence increased dramatically from grade 9 (21.0%) to grade 12 (50.8%) within the 2008 YSS [19], there is still a dramatic increase in the absolute number of youth using RYO as grade increases. Another finding consistent with research among adults [2,4,14], was that youth who reported more frequent marijuana use were more than twice as likely to currently use RYO tobacco. Considering the relatively common practice of users manually combining tobacco and marijuana (e.g., blunts) [20], future research should consider developing a better understanding of the link between RYO tobacco use and marijuana use among youth.

This study has several limitations common to survey research. Although the response rate was high and the data were weighted to help account for non-response, the findings are nevertheless subject to sample bias. It should also be noted that the cross-sectional nature of the design does not allow for causal inferences regarding the association between sociodemographic characteristics and RYO tobacco use. Longitudinal data are required. Moreover, our measure of RYO current use does not differentiate exclusive use from periodic use.

## Conclusions

RYO tobacco use has come to represent a small and shrinking market in Canada, but it is still responsible for tobacco related morbidity and mortality. Not only do youth RYO smokers tend to be heavier more addicted smokers, but they also tend to be younger, male, marijuana users, and have less disposable income than smokers who consume FM cigarettes. Considering that RYO use is prevalent among youth smokers in Canada, it is clear that the RYO market should not be ignored. Increasing the excise taxes (Federal and Provincial/Territorial taxes) on fine-cut tobacco used for RYO cigarettes to make the cost equivalent to FM cigarettes should be a public health priority. Ongoing research is needed to continue monitoring the prevalence of RYO use among youth and the factors associated with its use, but to also monitor if this more affordable tobacco product is being targeted to price sensitive youth smokers.

## Competing interests

The authors declare that they have no competing interests.

## Authors’ contributions

STL conceived the idea for the manuscript, developed the analysis plan, interpreted the results, wrote the manuscript, and prepared the manuscript for submission. RB performed the statistical analyses and helped develop the results section of the manuscript. Both authors read and approved the final manuscript.

## Pre-publication history

The pre-publication history for this paper can be accessed here:

http://www.biomedcentral.com/1471-2458/12/557/prepub
